# Supplementary material for: In vivo and ex vivo assessment of bladder hyper-permeability and using molecular targeted magnetic resonance imaging to detect claudin-2 in a mouse model for interstitial cystitis
Source: PLoS One. 2020 Oct 23;15(10):e0239282. doi: 10.1371/journal.pone.0239282 (PMC7584247; doi:10.1371/journal.pone.0239282)
Supplement: Attachment — Submitted filename: Response to Reviewers.docx [file pone.0239282.s002.docx]

**Response to Reviewers**

I would like to thank the Reviewers for their thoughtful suggestions and comments. We have addressed each comment below. The changes that we have made to the manuscript were substantial, and have dramatically improved the quality of the revised manuscript. Major changes include adding immunofluorescence data, adding an IgG isotype negative control for the molecular-targeting imaging data, quantification of all data, expanding the Methods section, and addition of relevant references.

Editor’s comments:

For the manuscript to be acceptable, please address the reviewer critiques as well as the following 6 points.

1. IHC staining may be acceptable if it can be demonstrated in images that claudin-2 is indeed at the tight junctions. If not, then IF should be considered because it may give a better signal, as suggested by reviewer.

*IF was included – new Figure 3.*

2. Quantification of all IHC and/or IF is required.

*Quantification for every figure has been included.*

3. For the figure 2 images, please consider whether there is an orientation difference or whether there is a difference in the nature of the umbrella cell layer. Consider choosing a better representative image if there are not overt histological differences.

*Better representative images have been included.*

4. Scale bars should be present in all microscopic images.

*Added as suggested.*

5. Check the labeling of Y axes. If it is percent, label as percent

*Changes as suggested.*

6. Avoid the word leaky when discussing claudin 2 and use the word hyperpermeability instead since it is a selectively permeable pathway.

*Changed.*

Reviewer #1:

1. A major concern is the specificity of the antibody-based probe towards claudin-2. A control with a probe with inactivated antibody, a probe with a control antibody or the biotin-albumin-Gd-DTPA should be performed.

*A non-specific IgG isotype contrast agent (replacing the claudin-2 antibody with a mouse IgG) has been added.*

2. Fig. 1 shows that using the Gd-DTPA probe the authors observe change in permeability between controls and LPS treated mice. Because the backbone of the antibody-based probe is Gd-DTPA, the question is whether this probe is binding to claudin-2 or just simply detecting changes in permeability.

*The probe is intravesically administered, but then washed out with saline after distilling in the bladder for an hour. Any residual probe would be washed out and not detected. The SA-HRP data clearly shows that the probe is present in the bladder urothelium.*

3. Please provide a detailed description of the animal model in the introduction, this will help the reader.

*Now included.*

4. Additional information should be provided about the method for coupling the claudin-2 antibody to the probe and the purification of the probe. Authors are encouraged to provide enough information so that the experiment can be duplicated. How was the mass of the probe determined?

*Additional information on the synthesis of the claudin-2 and IgG contrast agents has been included, along with an estimation of the molecular weight.*

5. Page 9, line 156. List the reagents produced by Vector Labs. State the concentration of claudin-2 Atb used.

*Updated and included.*

6. Number of animals and statistical test used should be indicated in the figure legends.

*Now included.*

7. Page 11, line 198. The images showed in Fig. 2 should be improved. It looks that the umbrella cell layer is gone in the LPS treated bladder. Is this correct? If possible, authors should quantify urothelial damage.

*We have used better representative images for Figure 2, as well as included quantification of Clnd2 expression. We have also additionally added trans-epithelial electrical resistance (TEER) data in Figure 1, that supports the increased permeability/damage to the LPS-induced mouse bladders, compared to saline controls.*

8. Claudin-2 only appears to be expressed in umbrella cells in control and across the whole urothelium in mice treated with LPS. Unfortunately, the immunohistochemical staining does not allow to define the cellular localization of claudin-2, which is relevant for its detection with the antibody-based probe. Authors should consider using immunofluorescence to assess whether Cldn2 is expressed in the plasma membrane and /or tight junctions.

*Immunofluorescence data has been added in a new figure, including quantification. From IF images it seems that Cldn2 is expressed in both the plasma membrane and tight junctions (added in figure legend – new Figure 3).*

9. The number in the panels of Fig.2 do not correspond with the figure legend.

*Changed.*

10. Page 13, last paragraph. The facts that the authors observe a signal by immunohistochemistry does not mean that the probe is detecting claudin-2 in the tissue. If the urothelium is leaky, the probe will diffuse and can be covalently attached to the tissue during fixation. This issue should be addressed.

*We are stating that we detect an increase in streptavidin-horse radish peroxidase (SA-HRP) which specifically targets the biotin moiety of either the claudin-2 probe or the IgG isotype control contrast agent. This can only occur if either probe or contrast agent is present. This has been shown to be the case for several probes using the Ab-albumin-Gd-DTPA-biotin construct by the authors (of which some of these references have been cited in this manuscript). We have included a non-specific IgG isotype contrast agent as a negative control. We have also rephrased the paragraph.*

11. Page 14, line 248. The statement is incorrect. There is no evidence that the overexpression of claudin-2 inhibits the synthesis of tight-junction proteins.

*Corrected.*

12. The increase in claudin-2 expression in mice treated with LPS should be quantified by western-blot.

*Quantification of the IHC, immunofluorescence, and molecular-targeted probe via streptavidin horse radish peroxidase which binds to the biotin moiety of the claudin-2 probe (and the non-specific IgG isotype contrast agent) was evaluated instead.*

Minor

13. Page 7, line 114. remove and retained for 10 min.

*Corrected.*

14. Please rephrase last sentence of the conclusions.

*Changed.*
