# Supplementary material for: In vivo and ex vivo assessment of bladder hyper-permeability and using molecular targeted magnetic resonance imaging to detect claudin-2 in a mouse model for interstitial cystitis
Source: PLoS One. 2020 Oct 23;15(10):e0239282. doi: 10.1371/journal.pone.0239282 (PMC7584247; doi:10.1371/journal.pone.0239282)
Supplement: Attachment — Submitted filename: Response to Reviewers 081920.docx [file pone.0239282.s003.docx]

**Response to Reviewers**

I would like to thank the Reviewers for their thoughtful suggestions and comments. We have changed the wording and included a Student’s t-test for data presented in Figs. 1, 2 and 3.

Reviewer #1:

The authors have addressed satisfactorily my previous comments.
An additional comment, t-test should be used for use to compare data from two groups (Fig. 1, 2 and 3). ANOVA is generally used to compare data from more than two groups.

*We have updated the statistical analyses and wording for Figs. 1-3 in the Methods and relevant figure legends. The p-values were not substantially changed, i.e. the asterisks reported for each figure remain the same, and therefore no changes were made to the figures.*
